# The Use of Evidence-Informed Deliberative Processes for Designing the Essential Package of Health Services in Pakistan

**DOI:** 10.34172/ijhpm.2023.8004

**Published:** 2023-10-24

**Authors:** Rob Baltussen, Maarten Jansen, Syeda Shehirbano Akhtar, Leon Bijlmakers, Sergio Torres-Rueda, Muhammad Khalid, Wajeeha Raza, Maryam Huda, Gavin Surgey, Wahaj Zulfiqar, Anna Vassall, Raza Zaidi, Sameen Siddiqi, Ala Alwan

**Affiliations:** ^1^Department of Health Evidence, Radboud University Medical Center, Nijmegen, The Netherlands.; ^2^Department of Health Services Policy and Management, Arnold School of Public Health, University of South Carolina, Columbia, SC, USA.; ^3^Department of Global Health & Development, London School of Hygiene and Tropical Medicine, London, UK.; ^4^Ministry of National Health Services, Regulations and Coordination, Islamabad, Pakistan.; ^5^Centre for Health Economics, University of York, York, UK.; ^6^Department of Community Health Sciences, Aga Khan University, Karachi, Pakistan.; ^7^DCP3 Country Translation Project, London School of Hygiene and Tropical Medicine, London, UK.

**Keywords:** Pakistan, Universal Health Coverage, Benefit Package, Priority Setting, Evidence-Informed Deliberative Process

## Abstract

**Background:** The Disease Control Priorities 3 (DCP3) project provides long-term support to Pakistan in the development and implementation of its universal health coverage essential package of health services (UHC-EPHS). This paper reports on the priority setting process used in the design of the EPHS during the period 2019-2020, employing the framework of evidence-informed deliberative processes (EDPs), a tool for priority setting with the explicit aim of optimising the legitimacy of decision-making in the development of health benefit packages.

**Methods:** We planned the six steps of the framework during two workshops in the Netherlands with participants from all DCP3 Pakistan partners (October 2019 and February 2020), who implemented these at the country level in Pakistan in 2019 and 2020. Following implementation, we conducted a semi-structured online survey to collect the views of participants in the UHC benefit package design about the prioritisation process.

**Results:** The key steps in the EDP framework were the installation of advisory committees (involving more than 150 members in several Technical Working Groups [TWGs] and a National Advisory Committee [NAC]), definition of decision criteria (effectiveness, cost-effectiveness, avoidable burden of disease, equity, financial risk protection, budget impact, socio-economic impact and feasibility), selection of interventions for evaluation (a total of 170), and assessment and appraisal (across the three dimensions of the UHC cube) of these interventions. Survey respondents were generally positive across several aspects of the priority setting process.

**Conclusion:** Despite several challenges, including a partial disruption because of the COVID-19 pandemic, implementation of the priority setting process may have improved the legitimacy of decision-making by involving stakeholders through participation with deliberation, and being evidence-informed and transparent. Important lessons were learned that can be beneficial for other countries designing their own health benefit package such as on the options and limitations of broad stakeholder involvement.

## Introduction

**Key Messages**

**Implications for policy makers**

- Pakistan recognises the importance of achieving universal health coverage (UHC), and the need for a revised essential package of health services (EPHS) to support this.
- In 2018, Pakistan initiated the development of a national EPHS by drawing on the Disease Control Priorities 3 (DCP3) evidence and approach, and implemented a national level priority setting process in 2019-2020.
- Implementation of the priority setting process resulted in a revised EPHS may have improved the legitimacy of the decision-making process and the decisions themselves by involving stakeholders through evidence-based deliberation and being transparent.

**Implications for the public**

This paper describes the process used by the government of Pakistan to establish the ‘Universal health coverage essential package of health services (UHC-EPHS)’ during the period 2019-2020. With this package of health services, the government aims to achieve UHC, ie, that all people have access to the full range of quality health services they need, when and where they need them, without financial hardship. The process involved a project team comprising scientists who collected evidence on eg, costs and health effects of the services. It also involved more than 150 stakeholders such as medical practitioners and policy-makers from different regions of the country, who interpreted this evidence and made recommendations to the government. This comprehensive decision-making process may contribute to the provision of health services that are most needed by the people of Pakistan.

Like many other countries around the world, Pakistan recognises the importance of an essential package of health services (EPHS) for achieving universal health coverage (UHC) and Sustainable Development Goal 3 (Good Health and Well-being).^1,2^ The country’s long-term development strategy for health (‘Vision 2025’) reiterates the need for a revised intervention package that offers protection against financial hardship to individuals and communities when accessing health interventions.^3^

 The Disease Control Priorities 3 (DCP3) initiative provides long-term support to Pakistan in the development and implementation of its EPHS. The DCP3 Country Translation project responds to the increasing need of low- and lower middle-income countries for technical guidance and support in benefit package design and in accelerating progress towards UHC.^4,5^ Pakistan is one of the first countries globally to implement the project.

 DCP3 provides a review of evidence on the efficacy, effectiveness and cost-effectiveness of interventions and a model benefit package for UHC. A priority setting process is needed to translate this evidence into EPHS design in Pakistan. Increasingly, decision-makers are urged to organise such processes in a fair, legitimate manner, with legitimacy referring to the reasonableness of decisions as perceived by stakeholders and being an important prerequisite for broad societal support.^6,7^

 Deliberative processes in healthcare are seen by many as a promising approach to achieve such legitimate decision-making for its merits in facilitating well-informed and inclusive decisions.^8-11^ Scholars have emphasized the advantages of incorporating diverse perspectives, evidence, and careful analysis into the decision-making process. Through open discussions and collaboration, the deliberative approach fosters stakeholder engagement, promoting a sense of ownership and acceptance of decisions, which enhances the likelihood of successful implementation. Potential drawbacks include its time-consuming nature and resource requirements, particularly in time-sensitive or resource-limited situations, difficulties in reaching consensus and risk of manipulation or dominance by powerful stakeholders.^9^ Deliberative processes are implemented by a range of governmental bodies in western countries that develop recommendations on the reimbursement of health interventions.^10^

 As part of a series on the DCP3 implementation in Pakistan, this paper reports on the priority setting process used in the development of the EPHS in Pakistan in the period 2019-2020, employing evidence-informed deliberative processes (EDPs). The EDP framework is a practical and stepwise tool for priority setting, with the dual aim to optimise the legitimacy of benefit package decisions, and related outcomes in terms of eg, population health and financial risk protection.^12-15^ In the framework, the concept of legitimacy is translated into four elements – stakeholder involvement, ideally operationalised through stakeholder participation with deliberation; evidence-informed evaluation; transparency; and appeal.^14^ The practical guidance on EDPs provides recommendations on how these elements can be implemented in each step of a decision-making process of EPHS design, based on a review of practices of countries around the world.^14^ Several other frameworks on priority setting are available,^7,16-19^ and the EDP framework can be considered complementary because of its explicit focus on stakeholder participation, including detailed practical guidance.

 A separate challenge for any priority setting process is how policy-makers can integrate benefit package decisions across the three axes of UHC, ie, to decide whether it should first expand the interventions package, improve population coverage for interventions, or reduce co-payments for interventions.^7^ This issue has been addressed within the EDP framework^13^ and this paper reports on its implementation in Pakistan.

 The research question in this paper is: how was the EDP framework implemented in the context of EPHS design in Pakistan and what is the stakeholders’ feedback on this process? The paper starts with the description of the process including the institutional context and the operationalisation of the EDP framework for EPHS design in Pakistan. We subsequently report on the survey used to assess stakeholder satisfaction with the priority setting process and survey results. We conclude by putting these results in a broader perspective. This paper is part of a series of papers on the development of the EPHS in Pakistan.^20-23^

## Description of the Priority Setting Process

###  Institutional Context

 The priority setting process was implemented by the Health Planning, System Strengthening and Information Analysis Unit of the Ministry of National Health Services, Regulations and Coordination (MNHSR&C), with technical support from the DCP3 Country Translation initiative, referred to as the UHC-EPHS Secretariat. Additional partners included the Community Health Sciences Department of Aga Khan University and Health Services Academy, London School of Hygiene & Tropical Medicine, World Health Organization (WHO) and Radboud University Medical Center (Radboudumc).

###  Operationalisation of the EDP Framework

**Figure 1:**
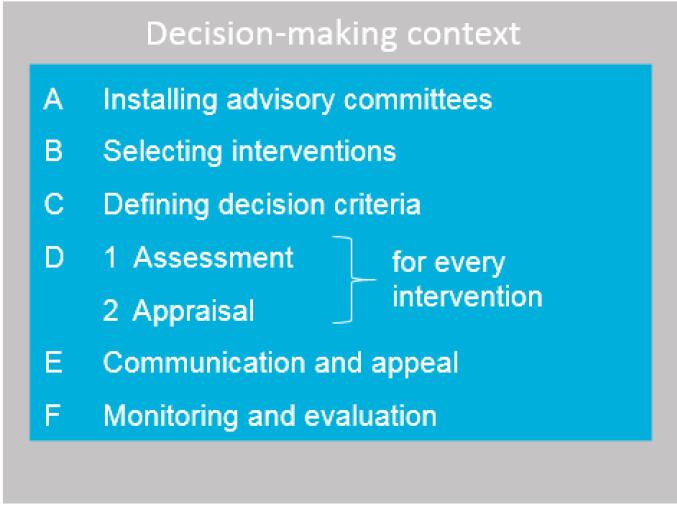
The Six Steps of the Evidence-Informed Deliberative Process Framework.

 We operationalised the steps of the EDP framework for the Pakistan context during two separate workshops at Radboudumc in the Netherlands, with participants from all DCP3 Pakistan project partners (October 2019 and February 2020). These steps are shown in Figure 1; of which steps A-D were realised during the present project and steps E-F are to be implemented in a subsequent stage. All procedures, templates and instructions were pilot tested and trainings for facilitators were organised prior to implementation at the UHC-EPHS workshops in Islamabad, Pakistan (November 2019 and February 2020).

###  Step A: Installing Advisory Committees

**Figure 2:**
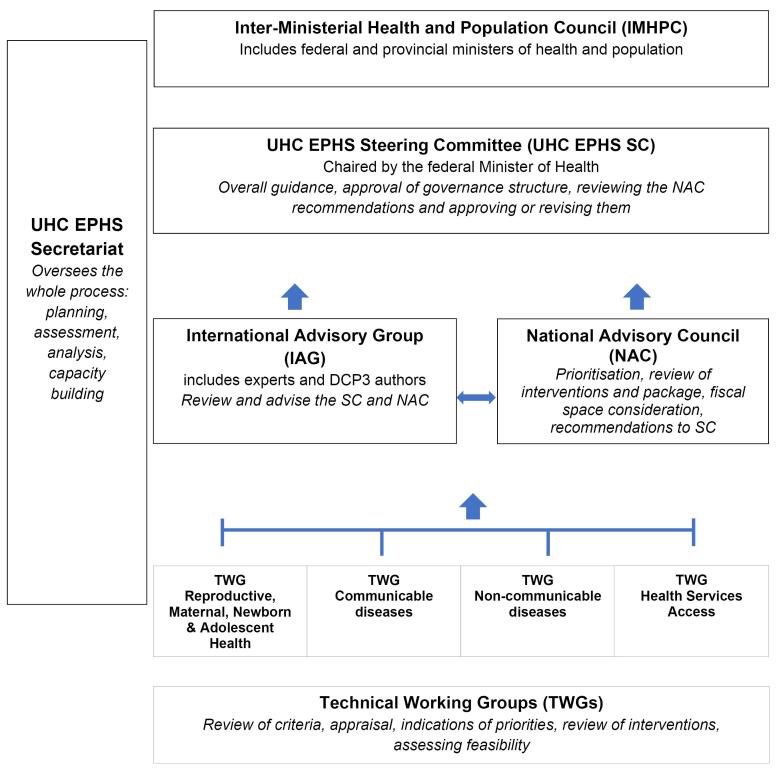
The Universal Health Coverage Essential Package of Health Services Governance Structure. Note: Arrows represent the flow of information. Reporting obligations are represented by larger arrows. Abbreviations: DCP3, Disease Control Priorities 3; EPHS, essential package of health services; UHC, universal health coverage.

 Supported by DCP3 partners, the UHC-EPHS Secretariat designed a governance structure for the EPHS based around three connected stages of deliberation around priorities (Figure 2).

 The first stage concerned the involvement of four already existing Technical Working Groups (TWGs) for specific disease clusters: reproductive, maternal, neonatal, child, and adolescent health (36 members), non-communicable diseases (38 members), communicable diseases (51 members), and health services access (26 members). These TWGs normally advise the MNHSR&C on various aspects of their respective disease areas, representing relevant stakeholders. This time they were tasked with reviewing the technical aspects of a range of interventions for potential inclusion into the UHC-EPHS, and broadly allocating services into priority categories.

 The second stage involved the establishment of a National Advisory Committee (NAC), whose mandate was to interpret the recommendations of the various TWGs and combine them into an overall set of recommendations. The NAC had 15 formal members.

 The third stage involved a high-level meeting of the Steering Committee (SC) responsible for reviewing the NAC recommendations and approving or revising them, and providing advice to the Minister of Health, who is responsible for making the final decision to implement the UHC-EPHS. An International Advisory Group (IAG) advised the SC. Terms of reference were drafted and adopted for each entity in the governance structure endorsed by the SC (shown in Figure 2). Conflict of interest declaration forms were drafted for completion by TWG and NAC members.

###  Step B: Selecting Interventions

 In a workshop in April 2019, the MNHSR&C, jointly with representatives from the provincial departments of health and other key stakeholders, reviewed the availability, coverage, and relevance of the DCP3 Essential UHC package (EUHC) in Pakistan. The DCP3 EUHC package is a model benefit package for UHC which lower- and middle-income countries are recommended to consider for the development of their national health benefit packages. Participants concluded that 170 (78%) out of the 218 EUHC services were relevant and feasible for Pakistan and should be assessed for possible inclusion in the UHC-EPHS, while others may be assessed and included at a later stage. None of the services currently provided in Pakistan were omitted at this stage.^24^

 Thereafter, the UHC-EPHS Secretariat further defined the selected DCP3 services in terms of processes and required resources, and the TWG members reviewed them before and during the UHC-EPHS workshops (November 2019 and February 2020).

###  Step C: Defining Decision Criteria

 In October-November 2019, the MNHSR&C conducted a survey on the use of decision criteria with the aim to develop consensus on the importance and definition of criteria for the prioritization of interventions by TWG and NAC members. It was sent electronically to all TWG and NAC members invited for the November meeting, with responses received from 52 members (response rate 52%). The following seven criteria were selected: effectiveness, burden of disease, health gain for money spent (defined as “cost-effectiveness” in the survey), equity, financial risk protection, budget impact, socio-economic impact, and feasibility. Details on the survey, its results and criteria definitions are provided in Supplementary file 1.

###  Step D1: Assessment 

 Assessment refers to the collection of evidence on the interventions considered. The UHC-EPHS Secretariat collected the available evidence on three criteria: budget impact (composed of individual intervention costs), cost-effectiveness, and burden of disease. Note that the criterion ‘burden of disease’ was used in the first meeting of the TWGs (TWG1) but was changed to ‘avoidable burden of disease by the intervention’ from the second meeting (TWG2) onwards as the latter criterion reflects both the burden of disease and the potential size of health gains. This was considered appropriate by the UHC-EPHS Secretariat, as burden of disease then directly relates to the intervention. This criterion then replaced the ‘Effectiveness’ criterion. The process of developing the evidence base is reported elsewhere.^20,22,23^ No quantitative evidence was collected for the remaining criteria for two reasons: effectiveness was not considered explicitly since all EUHC interventions were widely proven to be effective, while the others have insufficient data to make a quantitative assessment. These criteria were qualitatively assessed during the appraisal stage using expert opinion of the TWG members.

###  Step D2: Appraisal 

 Appraisal concerns the interpretation of the results of the assessment in a broader perspective and formulates a recommendation to inform decision-makers. This step involved the complex trade-off across the three UHC dimensions^13^ and was split into two sub-steps.

###  Appraisal Sub-step D2.1 – The Division of Interventions Into Priority Categories (Unconstrained by Fiscal Space)

**Figure 3:**
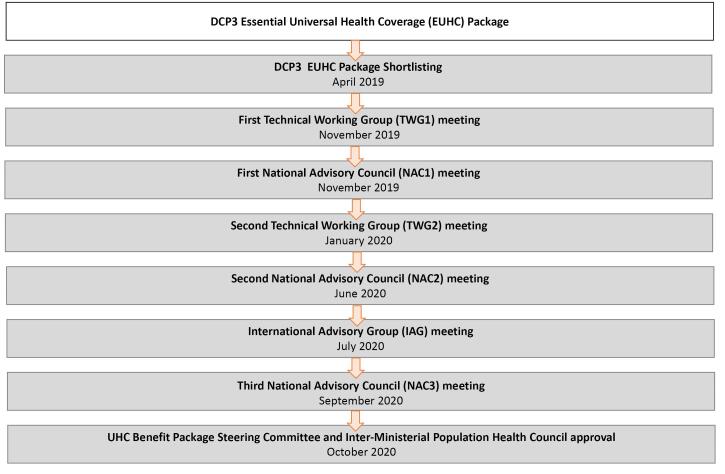
Timeline of Stages and Dates of the Appraisal Process. Abbreviations: DCP3, Disease Control Priorities 3; UHC, universal health coverage.

 The first sub-step involved the division of the 170 interventions into categories of ‘high priority,’ ‘medium priority’ and ‘low priority,’ reflecting their relative importance for the health system in Pakistan. To arrive at these categories, the TWGs interpreted the results of the assessment stage for each intervention and deliberated in Nov 2019 (TWG1 on community and primary care interventions) and in Feb 2020 (TWG2 on first level and referral hospital care) (see Figure 3). Each TWG covered between 28–51 interventions. At the meetings, the TWGs were split in smaller groups so each group could focus on smaller sets of around 10 interventions.

 Each TWG was allocated a trained facilitator, who had received instructions on how to follow a stepwise deliberative process. Each small group meeting started with an introduction, followed by a deliberative process for each intervention, which included reading of the intervention description, a round of clarification questions and answers, and an initial voting, in which each TWG members categorised the intervention as a high, medium or low priority. Based on their votes, the facilitator invited each TWG member to share his/her argumentation followed by group deliberation. Subsequently, members gave their last vote, and the rapporteur summarised the final voting results and argumentation. Several templates were available to facilitate this process, such as ‘evidence sheets’^13^ (also see Supplementary file 2, Figure S3) and ‘criteria explanation sheet.’ Voting was a crucial element of the deliberative process: it required TWG members to provide argumentation to justify their votes and thereby stimulated a more in-depth discussion; and it provided TWG members maximum influence on the decision-making process. To illustrate, Supplementary file 3 provides the instructions to the TWG facilitator on the deliberative process.

###  Appraisal Sub-step D2.2 – Making Choices Among High Priority Interventions (Accounting for Fiscal Space Constraints)

 Over the course of several meetings, the NAC subsequently reviewed TWG recommendations. An initial NAC meeting (NAC1) held in November 2019 reviewed TWG recommendations on community and health centre interventions. Supplementary file 2 provides the instruction to the NAC chair on the process. A second NAC meeting (NAC2), held in June 2022, had the complex task to further prioritise the list of interventions across all levels of the health system (community, health centre, primary hospitals, and referral hospitals) within the available fiscal space, taking into account coverage. To inform these decisions, the UHC-EPHS Secretariat prepared evidence on various packages with alternative assumptions on fiscal space, coverage levels, and co-payment levels; and taking into account the appropriate time horizon of NAC recommendations (0 to 10 years). Some packages also represented specific trade-offs, eg, explicitly prioritising high priority community interventions. During this second meeting, the NAC recommended two packages: a reduced package of interventions for immediate implementation (the immediate implementation package, or IIP) and a full EPHS to be implemented over a longer time horizon. The NAC recommendations on the IIP from the second meetings were reviewed by the IAG in July 2020, which suggested further changes. Lastly, the final iterations of both IIP and full EPHS were reviewed and approved by the UHC-EPHS SC and the Inter-Ministerial Health and Population Council in October 2020.

Figure 3 shows the timeline of the different stages of the appraisal process.

## Feedback on Process

###  Survey Design on Stakeholder Satisfaction

 We developed a semi-structured online survey to collect, ex-post, the views of EDP participants about the process that was followed. The survey was structured around the EDP steps (*i*) stakeholder involvement (reflecting the step ‘installing advisory committee’), (*ii*) selection of decision criteria, (*iii*) evidence collection (reflecting the step ‘assessment’), and (*iv*) appraisal. In addition, participants were asked about their satisfaction, acceptance and perceptions of the relevance of the process and its outcomes.

 For each theme, participants were asked to indicate to what extent they agreed with a set of statements using a five-point Likert scale. In addition, they were invited to respond to a series of open-ended questions on how these themes could be improved upon for future use. The survey was pretested with members of the DCP secretariat. Supplementary file 4 contains the survey questions.

###  Survey Results

 Out of 139 invited TWG/NAC members, 35 responded to the survey (25%). Respondents were overall positive about the process followed, with average scores for most questions of around 4 (+/-around 0.8) (Table). They were relatively critical (ie, here arbitrarily defined as items that received an average score of ≤ 3.7) as to whether (*i*) all important stakeholders were involved; (*ii*) there was sufficient time to understand the evidence; (*iii*) the evidence was sufficiently sensitive to the context of Pakistan; and (*iv*) the process and methods used had improved compared to previous approaches for HBP design in Pakistan. In their answers to open-ended questions, respondents mentioned, among others, they would value a more inclusive stakeholder involvement by inviting healthcare providers, clinicians, provincial representatives and ‘people working on the ground’; better communication in terms of document sharing and follow-up steps; better definitions and explanations of decision criteria; the inclusion of other criteria such as complementarity of interventions, and more attention for specific criteria such as feasibility; the use of local evidence and more transparency on evidence collection; a better stakeholder involvement in the appraisal phase. A thematic analysis of responses to open-ended questions is provided in Supplementary file 5.

**Table:** Views Regarding the Used Evidence-Informed Deliberative Process

**Topic**	**Statements **	**Average (Standard Deviation)** ^a^
Stakeholder involvement	It is clear to me how stakeholders were selected to participate in the EPHS design	3.9 (0.96)
	All important stakeholders were involved in the EPHS design	3.7 (1.09)
	My involvement in the EPHS design was valuable	4.0 (0.72)
	Involved stakeholders had equal opportunities to contribute during meetings	4.3 (0.77)
	Deliberation amongst stakeholders contributed to the development of my own opinions	4.1 (0.75)
	Views of involved stakeholders have been adequately taken into account in the EPHS design	4.2 (0.82)
	The NAC meeting in June 2020 was organized online, and this limited my understanding of the process	2.9 (1.06)^b^
	The NAC meeting in June 2020 was organized online, and this limited my involvement in the process	3.2 (1.11)^b^
The (use of) decision criteria	The criterion of ‘health gain for money spent’ was clear to me	4.1 (0.91)
	The criterion of ‘avoidable burden of disease by the intervention’ was clear to me	4.1 (0.83)
	The criterion of ‘budget impact’ was clear to me	4.1 (0.87)
	The criterion of ‘feasibility’ was clear to me	4.1 (0.81)
	The criterion of ‘equity’ was clear to me	4.1 (0.78)
	The criterion of ‘social and economic impact’ was clear to me	4.1 (0.91)
	The criterion of ‘financial risk protection’ was clear to me	4.0 (0.88)
	The decision criteria are an adequate reflection of the most important values for EPHS design	3.9 (0.89)
	The trade-offs between different criteria were clear to me	3.9 (0.90)
	Each criterion was adequately taken into account in the EPHS design	3.8 (0.91)
The (use of) evidence	The evidence presented was clear to me	3.9 (0.75)
	There was sufficient time to understand the evidence on each intervention	3.7 (0.87)
	The evidence presented was relevant to design the EPHS	3.9 (0.80)
	It is clear to me how the evidence was developed	3.8 (0.82)
	I am generally satisfied with the methods used to assess the evidence	3.8 (0.82)
	The evidence presented was sufficiently sensitive to the context of Pakistan	3.6 (0.90)
The appraisal process	There was sufficient time to deliberate on each intervention	3.9 (0.90)
	Each intervention was evaluated according to the same standards	4.1 (0.81)
	The process for taking decisions about the inclusion of interventions into the EPHS was clear to me	4.0 (0.70)
	I am satisfied with how decisions were taken about the inclusion of interventions in the EPHS	3.9 (0.85)
	The interventions under discussion were relevant to the context of Pakistan	4.1 (0.70)
Satisfaction, acceptance and relevance of the process and outcomes	The process and methods used have improved compared to previous approaches for EPHS design in Pakistan	3.4 (0.80)^c^
	The final content of the EPHS is acceptable for the context of Pakistan	4.0 (0.72)^c^
	I am satisfied with the outcomes of the EPHS process	3.9 (0.76)^c^
	The outcomes of the EPHS process are relevant to my setting/area	3.9 (0.75)^c^
	It is clear to me how the outcomes of the EPHS process will be used moving forward	1.9 (0.93)^c^

Abbreviations: NAC, National Advisory Committee; EPHS, Essential Package of Health Services. ^a^Scores reflect average responses to neutral questions on the listed themes, with responses ranging from 1 (‘fully disagree’) to 5 (‘fully agree’). ^b^Seven respondents were excluded (n = 28) due to only participating in the TWGs. ^c^One respondent was excluded (n = 34) due to self-reported inability to answer these questions.

## Discussion

 This paper described the implementation of the EDP framework in Pakistan and collected stakeholder feedback on this process. The use of EDPs may have contributed to the legitimacy of the UHC-EPHS decision process in Pakistan in three ways. First, the process involved more than 150 stakeholders through their participation as members in TWGs and/or the NAC. The meetings were organised in such a way that each member had equal chances to provide input in the deliberations and TWG members were granted voting power. In other words, stakeholders’ values were central in deliberations in various stages of the decision-making process. Second, the process was evidence-informed, ie, the discussions not only relied on formal evidence but also used expert judgements where relevant and necessary. TWG and NAC members brought in valuable expertise, especially on the practical implementation of interventions, and this added to the credibility of the decision-making process as a whole. Third, the use of EDPs contributed to transparency of decisions because final decisions were published, and the many involved stakeholders now have an understanding of the process. Our process evaluation survey indicates that stakeholders were generally and overall positive on these matters.

 The EPHS design process as described in this paper for the country of Pakistan shares many similarities with benefit package design/revision processes in other countries. A recent review of processes in Afghanistan, Ethiopia, Pakistan, Somalia, Sudan, and Zanzibar (Tanzania) identified that these countries follow largely the same steps – such as installing an advisory committee, selecting interventions for evaluation and selecting decision criteria – but also often differ in the practical operationalisation of the steps, eg, in the type of stakeholders that are being involved or in the choice of decision criteria.^25^ While there is no single ‘best practice’ in the operationalisation of these steps, countries’ experiences can inspire other countries in the design of their process. This was also the case in Pakistan where the EPHS process as described in this paper was based on existing local decision-making processes but also influenced by practices of other countries through the use of the EDP framework.^14^

 There were several practical challenges to the implementation of each of the steps of the EDP framework. Firstly, on the* advisory committees (step A)*, TWG and NAC members were generally satisfied with stakeholder involvement, but suggested participation could be improved in terms of its inclusive recruitment, specifically regarding representation by the provinces. In future applications of EDPs, efforts should be made to more proactively invite both sub-national stakeholders as well as public and patient representatives in advisory committees, or to elicit input from them through other means such as surveys. Broader stakeholder representation will further improve the legitimacy of the decision-making process, although it should be realised that this may involve higher organisational costs.

 In addition, not all TWG and NAC members may have the capacity to fully grasp the presented evidence. We did train members and provided them with instructions, criteria explanation sheets, evidence briefs and explicit rounds for clarification questions. This may have been successful – in our survey, TWG and NAC members reported to have a fairly good understanding of the criteria and related evidence although they also identified areas for improvement.

 Second, on the* decision criteria (step C)*, the UHC BP secretariat identified and selected a broad range of criteria through a stakeholder survey. Respondents were generally satisfied about the use of criteria, but also mentioned that these could be better defined and operationalized, such as ‘burden of disease avoided by the intervention.’ Although the project team pilot-tested criteria terminology and definitions among a sample of TWG and NAC members, this warrants more attention in future applications.

 Third, regarding the *assessment *of interventions (*step D1*), the Pakistan DCP team faced several challenges in compiling evidence. Due to both capacity and time constraints, the contextualisation of evidence using best practice translation methods was not always feasible, requiring the development of novel rapid ways of collating and analysing Pakistan specific data (as was the case with cost estimates)^20^ or translating international evidence to the local context (as was the case with cost-effectiveness data).^22^ While respondents generally agreed that the used methods to assess the evidence were acceptable, they mentioned that gathering more local evidence would be an improvement to the process.

 In addition, evidence is ideally collected on all decision criteria. In the current approach, the assessment of interventions in terms of their ‘feasibility,’ ‘equity,’ ‘financial risk protection,’ and ‘social and economic impact’ was based entirely on TWG and NAC members’ judgements as part of the appraisal process. This raises the risk that certain complex criteria, such as ‘equity’ were considered less thoroughly or objectively.

 Fourth, in the* appraisal *of interventions *(step D2)*, several aspects may have compromised the decision-making processes by the TWGs and NAC. Not all committee members had background knowledge to fully understand and interpret the presented evidence (eg, on cost-effectiveness of interventions). We did make efforts to instruct them with evidence briefs and explicit rounds for clarification questions, but it is not clear whether this was sufficient; while TWG and NAC members reported in our survey to have a good understanding of the evidence, we also observed that few committee members referred to evidence on cost-effectiveness, for example, to develop their judgment. In addition, several TWG and NAC members observed a shortage of expertise in some disease areas in their committees. Moreover, in the TWGs, time for appraisal was generally short, and only some 15-20 minutes were available for deliberation on each intervention. Furthermore, we observed that some facilitators and members were dominant during group meetings, which resulted in less balanced deliberations. These aspects are all known challenges to the use of deliberative process in healthcare decision-making.^8-11^ Nevertheless, TWG and NAC members were overall positive about the appraisal process.

 Fifth, the overall decision-making process was heavily disrupted by the COVID-19 pandemic. All TWG and NAC meetings were held on site until February 2020 but were conducted largely online afterwards. This may have compromised stakeholder participation, especially provincial engagement, and the quality of the decision-making process.

 An important limitation of our evaluation of the EDP implementation is the online survey that we conducted among participants. It resulted in broad notions of their satisfaction but precluded detailed insights into their experiences and perceptions of the strengths and weaknesses of the EDP framework. A more thorough approach might have revealed, for example, asymmetry of information and/or unequal power dynamics, but this would have required personal interviews and/or non-participant observations during the deliberations. Another limitation is the limited response (25%) to the online survey. We may have missed important perspectives from those who did not respond.

 An important contribution of this paper is how policy-makers can integrate benefit package decisions across the three axes of UHC.^7,13^ In the appraisal sub-step D2.1, decisions were made on the priority categories of interventions. In sub-step D2.2, fiscal space was considered, and decisions were made whether the intervention package should be expanded, or population coverage should be improved. To our knowledge, this is the first paper that has explicitly articulated these trade-offs into a set of scenarios and translated the complex interplay between benefit package design, UHC dimensions, and health system constraints into a series of practical steps. This aspect and other features of the EDP framework could serve as an example for other countries that wish to undertake health benefit package design or revision.

## Conclusion

 Despite several challenges, implementation of the priority setting process may have improved the legitimacy of decision-making by involving stakeholders through participation with deliberation and being evidence-informed and transparent. Important lessons were learned that can be beneficial for other countries designing their own health benefit package.

## Acknowledgements

 We express our gratitude to all TWG, NAC, and SC members who participated in the meetings and provided valuable feedback. In addition, we thank the IAG for providing constructive feedback on the materials and methods; and the WHO and the Bill & Melinda Gates Foundation for supporting this work.

## Ethical issues

 Ethical approvals were obtained from the London School of Hygiene and Tropical Medicine (21257) and Aga Khan University (2019-1992-5190); MoH clearance is being sought.

## Competing interests

 Authors declare that they have no competing interests.

## Funding

 This paper is part of a series of papers coordinated by the DCP3 Country Translation Project at the London School of Hygiene and Tropical Medicine, which is funded by the Bill & Melinda Gates Foundation [Grant OPP1201812]. The sponsor had no involvement in paper design; collection, analysis and interpretation of the data; and in the writing of the paper.

## Supplementary files


Supplementary file 1. Survey on Decision Criteria and Presentation Sheet.

Supplementary file 2. Appraisal Sub-step D2.2 – Instructions for the NAC Chair.

Supplementary file 3. Appraisal Sub-step D2.1 – Instructions for the TWG Facilitator.

Supplementary file 4. Priority Setting Process Evaluation Survey.

Supplementary file 5. Full Results of Priority Setting Process Evaluation Survey.

## References

[1] United Nations. Sustainable Development Goals. Goal 3: Ensure Healthy Lives and Promote Well-Being for All at All Ages. 2016. http://www.un.org/sustainabledevelopment/health/.

[2] Government of Pakistan. National Initative for Sustainable Development Goals. https://www.sdgpakistan.pk/.

[3] Planning Commission Ministry of Planning, Development & Reform. Pakistan 2025: One Nation - One Vision. Islamabad. https://www.pc.gov.pk/uploads/vision2025/Pakistan-Vision-2025.pdf.

[4] Disease Control Priorities Project, Third Edition. University of Washington. http://dcp-3.org/.

[5] Jamison DT, Alwan A, Mock CN (2018). Universal health coverage and intersectoral action for health: key messages from Disease Control Priorities, 3rd edition. Lancet.

[6] Daniels N (2000). Accountability for reasonableness. BMJ.

[7] World Health Organization (WHO). WHO Consultative Group on Equity and Universal Health Coverage. Making Fair Choices on the Path to UHC. Geneva: WHO; 2016.

[8] Abelson J, Forest PG, Eyles J, Smith P, Martin E, Gauvin FP (2003). Deliberations about deliberative methods: issues in the design and evaluation of public participation processes. Soc Sci Med.

[9] Safaei J (2015). Deliberative democracy in health care: current challenges and future prospects. J HealthcLeadersh.

[10] Bond K, Stiffell R, Ollendorf DA. Principles for deliberative processes in health technology assessment. Int J Technol Assess Health Care. 2020:1-8. 10.1017/s0266462320000550. 32746954

[11] Oortwijn W, Husereau D, Abelson J (2022). Designing and implementing deliberative processes for health technology assessment: a good practices report of a joint HTAi/ISPOR task force. Value Health.

[12] Baltussen R, Jansen MPM, Bijlmakers L (2017). Value assessment frameworks for HTA agencies: the organization of evidence-informed deliberative processes. Value Health.

[13] Baltussen R, Jansen MP, Bijlmakers L, Tromp N, Yamin AE, Norheim OF (2017). Progressive realisation of universal health coverage: what are the required processes and evidence?. BMJ Glob Health.

[14] Oortwijn W, Jansen M, Baltussen R. Evidence-Informed Deliberative Process: A Practical Guide for HTA Bodies for Legitimate Benefit Package Design. Nijmegen: Radboud University Medical Center; 2021. https://www.radboudumc.nl/global-health-priorities.

[15] Oortwijn W, Jansen M, Baltussen R (2022). Evidence-informed deliberative processes for health benefit package design - part II: a practical guide. Int J Health Policy Manag.

[16] Terwindt F, Rajan D, Soucat A. Priority-setting for national health policies, strategies and plans. In: Strategizing National Health in the 21st Century: A Handbook. Geneva: World Health Organization; 2016.

[17] Glassman A, Giedion U, Smith PC. What’s In, What’s Out: Designing Benefits for Universal Health Coverage. Washington, DC: Brookings Institution Press, Center for Global Development; 2017.

[18] International Decision Support Initiative (iDSI). The HTA Toolkit. 2018. Available from: http://www.idsihealth.org/HTATOOLKIT/.

[19] Castro H, Suharlim C, Kumar R. Moving LMICs Toward Self-Reliance: A Roadmap for Systematic Priority Setting for Resource Allocation. Available from: https://msh.org/resources/a-roadmap-for-systematic-priority-setting-and-health-technology-assessment-hta-a-practical/.

[20] Raza W, Zulfiqar W, Shah MM, et al. Costing interventions for developing an Essential Package of Health Services: application of a rapid method and results from Pakistan. Int J Health Policy Manag 2023; Forthcoming. 10.34172/ijhpm.2023.8006 PMC11607593 39099514

[21] Torres-Rueda S, Vassall A, Zaidi R, et al. The use of evidence to design an Essential Package of Health Services in Pakistan: a review and analysis of prioritisation decisions at different stages of the appraisal process. BMJ Glob Health 2023; Forthcoming. 10.34172/ijhpm.2024.8043 PMC11608344 39099513

[22] Huda M, Kitson N, Saadi N, et al. Assessing global evidence on cost-effectiveness to inform development of Pakistan’s Essential Package of Health Services. Int J Health Policy Manag 2023; Forthcoming. 10.34172/ijhpm.2023.8005 PMC11607590 39099515

[23] Alwan A, Siddiqi S, Safi M, et al. Addressing the UHC challenge using the Disease Control Priorities-3 approach: learning from the Pakistan experience. Int J Health Policy Manag 2023; Forthcoming. 10.34172/ijhpm.2023.8003 PMC11607589 39099517

[24] Ministry of National Health Services, Regulations and Coordination. Review of Essential Health Services in Pakistan Based on Disease Control Priorities-3. April 2019. Available at: http://www.nhsrc.gov.pk/.

[25] Baltussen R, Mwalim O, Blanchet K (2023). Decision-making processes for essential packages of health services: experience from six countries. BMJ Glob Health.

