# Supplementary material for: The Use of Evidence-Informed Deliberative Processes for Designing the Essential Package of Health Services in Pakistan
Source: Int J Health Policy Manag. 2023 Oct 24;12:8004. doi: 10.34172/ijhpm.2023.8004 (PMC10699818; doi:10.34172/ijhpm.2023.8004)
Supplement: Supplementary file 1 — Survey on Decision Criteria and Presentation Sheet. [file ijhpm-12-8004-s001.pdf]

**Article title:** The Use of Evidence-Informed Deliberative Processes for Designing the Essential Package of Health Services in Pakistan

**Journal name:** International Journal of Health Policy and Management (IJHPM)

**Authors' information:** Rob Baltussen[1]*¶, Maarten Jansen[1]¶, Syeda Shehirbano Akhtar[2], Leon Bijlmakers[1], Sergio Torres-Rueda[3], Muhammad Khalid[4], Wajeeha Raza[5], Maryam Huda[6], Gavin Surgey[1], Wahaj Zulfiqar[4], Anna Vassall[3], Raza Zaidi[4], Sameen Siddiqi[6], Ala Alwan[7]

[1]Department of Health Evidence, Radboud University Medical Center, Nijmegen, The Netherlands.

[2]Department of Health Services Policy and Management, Arnold School of Public Health, University of South Carolina, Columbia, SC, USA.

[3]Department of Global Health & Development, London School of Hygiene and Tropical Medicine, London, UK.

[4]Ministry of National Health Services, Regulations and Coordination, Islamabad, Pakistan.

5Centre for Health Economics, University of York, York, UK.

[6]Department of Community Health Sciences, Aga Khan University, Karachi, Pakistan.

[7]DCP3 Country Translation Project, London School of Hygiene and Tropical Medicine, London, UK.

***Correspondence to:** Rob Baltussen; Email: Rob.Baltussen@Radboudumc.nl

**Citation:** Baltussen R, Jansen M, Akhtar SS, et al. The use of evidence-informed deliberative processes for designing the essential package of health services in Pakistan. Int J Health Policy Manag. 2023;12:8004. doi:10.34172/ijhpm.2023.8004

**Supplementary file 1.** Survey on Decision Criteria and Presentation Sheet

**Supplementary file 1. Survey on Decision Criteria and Explanation Sheet**

With support of the UHC EPHS Secretariat and Radboudumc, the Ministry organized a survey on decision criteria in Oct-Nov 2019. The aim of the survey was to develop consensus on the importance and definition of criteria for the prioritisation of interventions to guide TWG members as they categorize interventions as high, medium or low priority. It was submitted electronically to all TWG and NAC members invited for the Nov meeting using Google Forms.

The survey asked respondents to indicate the importance they attached to pre-defined criteria for prioritizing interventions for inclusion into the UHC benefit package, whether they believed any criteria were missing, and to provide any additional comments or suggestions.

The pre-selection of eight criteria included in the survey (health gain for money spent, effectiveness, burden of disease, budget impact, feasibility, equity, financial risk protection, and social and economic impact) was based on the Ministry's initial scoping exercise (see below) and a subsequent document review of relevant UHC policy documents provided by the Ministry to identify the criteria they refer to in relevant policy documents. Subsequently, the identified criteria were matched to the criteria proposed in the literature, for which a recent extensive review of decision criteria was used.[1] Finally, during the first workshop in Nijmegen (Oct 2019) a subgroup with representatives from the Ministry, LSHTM and Radboudumc further specified the criteria and their definitions for feedback and approval by TWG members. The survey is shown in Figure S1.

In total, 52 invited TWG members responded to the Likert-scale survey (response rate 52%). Based on the survey results, and feedback following the first appraisal workshop, several of the criteria were redefined (mainly phrased more in laymen's language). Especially the cost-effectiveness criterion proved difficult for participants to grasp and was rephrased as 'health gain for money spent'. No additional criteria were suggested. While effectiveness was one of the original criteria it was not used during the prioritisation exercise as the interventions subjected to deliberation and prioritisation were all considered effective, being a requirement for their inclusion in the DCP3 list of recommended interventions. Figure S2 and Table S1 show the results, and Table S2 presents the used decision criteria and their definitions.

[1] Rehfuess EA, Stratil JM, Scheel IB, et al. The WHO-INTEGRATE evidence to decision framework version 1.0: integrating WHO norms and values and a complexity perspective. BMJ Global Health 2019;4:e00084

**Figure S1.** Survey on decision criteria

For the attention of all those who will take part in the meeting 'Development of EPHS/UHC-EPHS for Pakistan', end of November.

The Ministry of National Health Services, Regulations & Coordination and the DCP3 secretariat would be pleased to receive your responses to a couple of questions about how to set priorities for the health sector in Pakistan. Your answers will help us (organisers of the above meeting) to prepare ourselves. At the same time, the questionnaire will give you an idea of the type of deliberations that we will be having during the meeting.

Completion of the questionnaire will take you about 10 minutes. We will observe confidentiality, which means that when we present the results of this survey it will not be able to link answers to individual respondents. By agreeing to complete the questionnaire you will accept these conditions.

Most of the questions below are about reasons (criteria) to include or exclude interventions from the essential package of health services.

Please read carefully through each of the following criteria and their definitions.

| Criteria: | Definitions: |
|---|---|
| Effectiveness | The balance of health benefits and harms that reflects the health impact of an intervention on individuals or populations. |
| Burden of Disease | The health loss from diseases, injuries and risk factors at the population level; it is usually expressed as a measure that combines morbidity, mortality and disability. |
| Feasibility | The extent to which the intervention can be delivered through the existing health system taking into account available human resources, infrastructure and other resources and whether it is socio-culturally acceptable to the public. |
| Cost-effectiveness | The value-for-money of the intervention; usually expressed as a ratio of the costs of the intervention to its benefits. |
| Equity | The extent to which an intervention gives priority to the worse-off in terms of health-status, socio-economic status and/or service coverage. |
| Budget impact | The overall financial implications of implementing the intervention for the available national health budget. |
| Financial risk protection | The extent to which individuals, households or communities can afford the cost of the intervention and are protected from catastrophic health expenditure and health-related financial risk. |
| Social and economic impact | The societal consequences resulting from the intervention, for instance in terms of stigma, societal cohesion; as well as the broader economic consequences, such as national development and poverty reduction goals. |

1. Q1: From your PERSONAL point of view, how important do you consider each of the following criteria for prioritization of interventions in the EPHS? *

| | Not important | 2 | 3 | 4 | Very important |
|---|---|---|---|---|---|
| **Effectiveness** | O | O | O | O | O |
| **Burden of disease** | O | O | O | O | O |
| **Feasibility** | O | O | O | O | O |
| **Cost-effectiveness** | O | O | O | O | O |
| **Equity** | O | O | O | O | O |
| **Budget impact** | O | O | O | O | O |
| **Financial risk protection** | O | O | O | O | O |
| **Social and economic impact** | O | O | O | O | O |

2. Please provide your comments, if you have any, on the above criteria
3. Are there other criteria that YOU consider important to prioritize interventions in the EPHS? If yes, which ones?

**Criteria survey results:**

**Figure S2.** Results of survey on decision criteria: distribution of responses to the question: ‘From your PERSONAL point of view, how important do you consider each of the following criteria for prioritization of interventions in the EPHS?’ – using a Likert scale from 1 (not important) to 5 (very important).

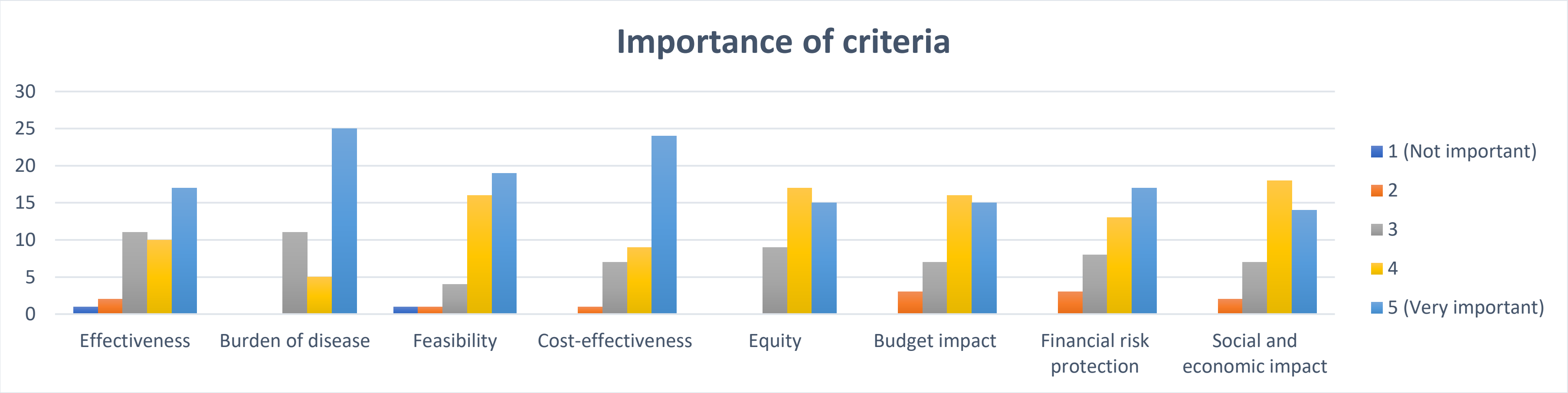

**Table S1.** Results of survey on decision criteria: average scores

| | | |
|---|---|---|
| 1. | Cost-effectiveness | 4.4 |
| 2. | Burden of disease | 4.3 |
| 3. | Feasibility | 4.2 |
| 4. | Equity | 4.1 |
| 5. | Financial risk protection | 4.1 |
| 6. | Social and economic impact | 4.1 |
| 7. | Budget impact | 4.1 |
| 8. | Effectiveness | 4.0 |

**Table S2:** Criteria explanation sheet

| Criteria: | Definitions: | Operationalization (indicating low (red), medium (amber) or high (green) performance): | | |
|---|---|---|---|---|
| **1. Health gain for money spent** | The cost-effectiveness of the intervention; expressed as a ratio of the costs of the intervention to its health gains for the population. The rank-order among the total of 83 interventions is also provided (rank 1/83 = most health gain for money spent). | Low health gains for PKR | Medium health gains for PKR | High health gains for PKR |
| 2. **Avoidable burden of disease (BoD) by the intervention*** | The health loss of a disease which can be avoided by the intervention. | Low avoidable BoD | Medium avoidable BoD | High avoidable BoD |
| **3. Budget impact** | The overall financial implications of implementing the intervention for the national health budget. The budget impact is presented as share of the total budget. | The intervention uses more than 1% of budget | The intervention uses between 0.5% - 1% of budget | The intervention uses less than 0.5% of budget |
| **4. Feasibility** | The extent to which an intervention can be delivered through the existing health system taking into account e.g. available human resources and infrastructure, and whether it is socio-culturally acceptable to the public. | N.A. | | |
| **5. Equity** | The extent to which an intervention targets vulnerable groups, e.g. the severely ill, the poor, certain ethnic groups, children or women. This may be a reason to prioritize such an intervention for public funding. | N.A. | | |
| **6. Financial risk protection** | The extent to which inclusion into the package protects individuals against costs related to the disease or accessing the intervention. This may be a reason to prioritize such an intervention for public funding. | N.A. | | |
| **7. Social and economic impact** | The extent to which an intervention results in societal consequences, e.g. in terms of stigma; as well as the broader economic | N.A. | | |

| | | | | |
|---|---|---|---|---|
| | consequences, such as national development. This may be a reason to prioritize such an intervention for public funding. | | | |
| ***Applicability of the evidence to Pakistan*** | | ☆<br><br>No evidence available or not possible to review | ☆ ☆<br><br>Evidence partially matches* | ☆☆☆<br><br>Local evidence that matches the intervention |

**The criterion avoidable burden of disease replaced burden of disease and was used by TWG2 onwards.*

*** A partial evidence match could mean that the evidence had an i) exact intervention match and partial geographic match, or ii) a partial intervention match and partial geographic match.*
