# Supplementary material for: The Use of Evidence-Informed Deliberative Processes for Designing the Essential Package of Health Services in Pakistan
Source: Int J Health Policy Manag. 2023 Oct 24;12:8004. doi: 10.34172/ijhpm.2023.8004 (PMC10699818; doi:10.34172/ijhpm.2023.8004)
Supplement: Supplementary file 2 — Appraisal Sub-step D2.2 – Instructions for the NAC Chair. [file ijhpm-12-8004-s002.pdf]

Article title: The Use of Evidence-Informed Deliberative Processes for Designing the Essential Package of Health Services in Pakistan

Journal name: International Journal of Health Policy and Management (IJHPM)

Authors' information: Rob Baltussen[1]*¶, Maarten Jansen[1]¶, Syeda Shehirbano Akhtar[2], Leon Bijlmakers[1], Sergio Torres-Rueda[3], Muhammad Khalid[4], Wajeeha Raza[5], Maryam Huda[6], Gavin Surgey[1], Wahaj Zulfiqar[4], Anna Vassall[3], Raza Zaidi[4], Sameen Siddiqi[6], Ala Alwan[7]

[1]Department of Health Evidence, Radboud University Medical Center, Nijmegen, The Netherlands.

[2]Department of Health Services Policy and Management, Arnold School of Public Health, University of South Carolina, Columbia, SC, USA.

[3]Department of Global Health & Development, London School of Hygiene and Tropical Medicine, London, UK.

[4]Ministry of National Health Services, Regulations and Coordination, Islamabad, Pakistan.

5Centre for Health Economics, University of York, York, UK.

[6]Department of Community Health Sciences, Aga Khan University, Karachi, Pakistan.

[7]DCP3 Country Translation Project, London School of Hygiene and Tropical Medicine, London, UK.

***Correspondence to:** Rob Baltussen; Email: Rob.Baltussen@Radboudumc.nl

Citation: Baltussen R, Jansen M, Akhtar SS, et al. The use of evidence-informed deliberative processes for designing the essential package of health services in Pakistan. Int J Health Policy Manag. 2023;12:8004. doi:10.34172/ijhpm.2023.8004

Supplementary file 2. Appraisal Sub-step D2.2 – Instructions for the NAC Chair

As the NAC Chair you are charged with supporting the NAC members in arriving at a draft recommendation on UHC/BP interventions.

Introduction

The Chair will firstly provide the NAC members with a general introduction of what is expected of them and how the NAC will achieve this. Points to emphasize:

- Over the past 2 days, the 4 TWGs (divided in a total of 8 groups) have prioritized interventions into low/medium/high priority 'buckets'
- A consolidated rank-ordering of interventions is being created for each of the low/medium/high priority buckets based on the voting results of the TWGs
- The aim of the NAC meeting is to further review part of this consolidated rank-ordering and adjust it – where appropriate – by identifying interventions that should be 'in' or 'out' of the EPHS supported by the necessary argumentation
- The agenda for day 3 and 4 is as outlined below

Proceedings for day 3 (first day of NAC meeting)

i. During the morning of day 3 you will invite each of the TWG representatives on the NAC to briefly present their TWG recommendations (8 groups, 10-15 mins each).
    a. TWG representatives will present which of their respective interventions are in each priority bucket and the main argumentation
        i. For each of the TWGs the project team will provide 2-3 template PowerPoint slides specifying which of the interventions are given high/medium/low priority by TWG participants – based on majority votes
        ii. Presentations on the high and low priority buckets will be short; the presentation of medium priority buckets can be a bit more elaborate.

Proceedings for day 4 (second day of NAC meeting)

i. At the start of day 4 all NAC members will receive a printed version of the combined rank-ordering of interventions, created by combining all TWG recommendations obtained into one overall rank-ordering. The budget limit will have been added to this rank-ordering.
    a. Discussions will focus on inclusion or exclusion of medium-priority interventions, starting with the highest ranked intervention, followed by the second highest ranked intervention and so on, down to the lowest ranked intervention in the medium-priority class.
ii. As the NAC Chair you will work towards formulating recommendations on each of the interventions in the medium-priority bucket. For each intervention:

a. You first invite the TWG representative to summarize the argumentations used by TWG participants
b. Then you invite NAC members to provide additional argumentations to either:
    i. Include the intervention in the EPHS
    ii. Exclude the intervention from the EPHS
    iii. Defer the decision on inclusion/exclusion to a later point in time in case the available evidence is insufficient to reach a justifiable decision
c. When no (more) arguments are provided, or time is up, you will call for a vote on whether the intervention should be (i) Included, (ii) Excluded or (iii) Deferred.
d. Depending on the voting results there will be four possible recommendations:
    i. Include the intervention if voting is (almost) unanimous in favor of inclusion
    ii. Exclude the intervention if voting is (almost) unanimous in favor of exclusion
    iii. Defer the recommendation if the available evidence is insufficient
    iv. Defer the recommendation if the available evidence appears sufficient but there is no clear majority for either inclusion or exclusion.

iii. As a final step, the NAC recommendations will be used to further narrow down the list of interventions recommended for inclusion into the EPHS.

Figure S3: Evidence sheets

Service # and name:

**Health gain for money spent**	
Medium health gains for PKR spent<br><br>44/86	*Applicability of the evidence to Pakistan*<br><br>★ ★ ☆

**Budget impact**
<0.5% of budget<br><br>0.2 PKR per capita

**Avoidable BoD by the intervention**
Low avoidable BoD

Equity: *Does it target vulnerable groups?*

Financial risk protection: *Does it protect against financial risk?*

Social and economic impact: *Does it have broader socio-economic consequences?*

Feasibility: *Can it be delivered and is it socio-culturally acceptable?*
