# Supplementary material for: The Use of Evidence-Informed Deliberative Processes for Designing the Essential Package of Health Services in Pakistan
Source: Int J Health Policy Manag. 2023 Oct 24;12:8004. doi: 10.34172/ijhpm.2023.8004 (PMC10699818; doi:10.34172/ijhpm.2023.8004)
Supplement: Supplementary file 3 — Appraisal Sub-step D2.1 – Instructions for the TWG Facilitator. [file ijhpm-12-8004-s003.pdf]

Article title: The Use of Evidence-Informed Deliberative Processes for Designing the Essential Package of Health Services in Pakistan

Journal name: International Journal of Health Policy and Management (IJHPM)

Authors' information: Rob Baltussen[1]*¶, Maarten Jansen[1]¶, Syeda Shehirbano Akhtar[2], Leon Bijlmakers[1], Sergio Torres-Rueda[3], Muhammad Khalid[4], Wajeeha Raza[5], Maryam Huda[6], Gavin Surgey[1], Wahaj Zulfiqar[4], Anna Vassall[3], Raza Zaidi[4], Sameen Siddiqi[6], Ala Alwan[7]

[1]Department of Health Evidence, Radboud University Medical Center, Nijmegen, The Netherlands.

[2]Department of Health Services Policy and Management, Arnold School of Public Health, University of South Carolina, Columbia, SC, USA.

[3]Department of Global Health & Development, London School of Hygiene and Tropical Medicine, London, UK.

[4]Ministry of National Health Services, Regulations and Coordination, Islamabad, Pakistan.

5Centre for Health Economics, University of York, York, UK.

[6]Department of Community Health Sciences, Aga Khan University, Karachi, Pakistan.

[7]DCP3 Country Translation Project, London School of Hygiene and Tropical Medicine, London, UK.

***Correspondence to:** Rob Baltussen; Email: Rob.Baltussen@Radboudumc.nl

Citation: Baltussen R, Jansen M, Akhtar SS, et al. The use of evidence-informed deliberative processes for designing the essential package of health services in Pakistan. Int J Health Policy Manag. 2023;12:8004. doi:10.34172/ijhpm.2023.8004

Supplementary file 3. Appraisal Sub-step D2.1 – Instructions for the TWG Facilitator

As the facilitator you are charged with supporting the TWG in coming to a draft recommendation on UHC-EPHS interventions. For each intervention the group must prepare its recommendation within 30-45 mins.

The rapporteur is charged with filling in the TWG-reporting sheet and assisting the facilitator in memorizing the course of the deliberations. In case you are not sure how to record certain arguments brought forward by participants you need to check with the facilitator. After completion of the deliberation for each intervention you will summarize the results to the group for validation.

By way of introduction, the facilitator starts by providing the TWG participants with a general overview of what is expected of them and the procedure that will be followed. Please explain:

- The aim is to receive recommendations from TWG participants on whether the listed interventions should be considered high/medium/low priority and to obtain their argumentations
    - **High priority** interventions should be recommended for inclusion in the package
    - **Medium priority** interventions should only be recommended for inclusion in the package after the high priority interventions have been implemented and there is sufficient budget to do so.
        - Interventions can also be recommended as medium priority based on certain conditions that require further discussion by the NAC. They may be included but implementation deferred, because of:
            1. insufficient evidence, further local research or piloting is required
            2. there is evidence, but for other reasons the intervention should not be scaled-up immediately, e.g. very unfeasible
            3. the platform of the intervention should be changed first
            4. there is an issue with the name or content of the intervention
    - **Low priority** interventions should be only be considered after all high and medium priorities are implemented or they should not be considered at all.
- The number of interventions to be handled by your TWG varies between … and …
- The available time per intervention: 30-45 mins
- The proceedings for each intervention appraisal round are as outlined in the textbox below

- Documents that will be provided are:
    1. Booklet with intervention descriptions and evidence summaries
    2. Booklet to write down argumentation.
    3. Explanations of criteria

Proceedings for each intervention appraisal round

i. Instruct each participant to read the intervention description themselves

ii. Present the evidence summary of the intervention to participants

iii. Check if there are any questions for clarification (do not allow participants to already express their own preferences or priorities)

iv. Instruct participants to write down their first vote (high/medium/low priority) and their argumentation in their argumentation notebooks

v. Check if everyone is ready to vote and proceed to voting

vi. After the vote, systematically invite each participant to share their argumentation:
   - Start by asking participants who voted high priority to share their arguments
   - Second, ask participants who voted medium priority to share their arguments
   - Thirdly, ask participants who voted low priority to share their arguments
   - Fourthly, invite participants to deliberate amongst each other. Deliberations are finished when no more arguments are provided or time is up.
   - Fifthly, ask participants to reflect on any criteria that have not yet been put forward

vii. Call for a final round of votes on the priority class (high/medium/low)

viii. Ask the rapporteur to summarize the recommendation for validation by the group:
   a. the final voting results
   b. the final argumentation provided for each priority class

ix. The facilitator then closes the discussion and moves on to introduce the next intervention.

START: please follow for each intervention the proceedings as detailed in the box above.
