# Supplementary material for: The Use of Evidence-Informed Deliberative Processes for Designing the Essential Package of Health Services in Pakistan
Source: Int J Health Policy Manag. 2023 Oct 24;12:8004. doi: 10.34172/ijhpm.2023.8004 (PMC10699818; doi:10.34172/ijhpm.2023.8004)
Supplement: Supplementary file 4 — Priority Setting Process Evaluation Survey. [file ijhpm-12-8004-s004.pdf]

Article title: The Use of Evidence-Informed Deliberative Processes for Designing the Essential Package of Health Services in Pakistan

Journal name: International Journal of Health Policy and Management (IJHPM)

Authors' information: Rob Baltussen[1]*¶, Maarten Jansen[1]¶, Syeda Shehirbano Akhtar[2], Leon Bijlmakers[1], Sergio Torres-Rueda[3], Muhammad Khalid[4], Wajeeha Raza[5], Maryam Huda[6], Gavin Surgey[1], Wahaj Zulfiqar[4], Anna Vassall[3], Raza Zaidi[4], Sameen Siddiqi[6], Ala Alwan[7]

[1]Department of Health Evidence, Radboud University Medical Center, Nijmegen, The Netherlands.

[2]Department of Health Services Policy and Management, Arnold School of Public Health, University of South Carolina, Columbia, SC, USA.

[3]Department of Global Health & Development, London School of Hygiene and Tropical Medicine, London, UK.

[4]Ministry of National Health Services, Regulations and Coordination, Islamabad, Pakistan.

5Centre for Health Economics, University of York, York, UK.

[6]Department of Community Health Sciences, Aga Khan University, Karachi, Pakistan.

[7]DCP3 Country Translation Project, London School of Hygiene and Tropical Medicine, London, UK.

***Correspondence to:** Rob Baltussen; Email: Rob.Baltussen@Radboudumc.nl

Citation: Baltussen R, Jansen M, Akhtar SS, et al. The use of evidence-informed deliberative processes for designing the essential package of health services in Pakistan. Int J Health Policy Manag. 2023;12:8004. doi:10.34172/ijhpm.2023.8004

Supplementary file 4. Priority Setting Process Evaluation Survey

Welcome to the survey on the development of the universal health coverage benefit-package (HBP) to which you have contributed as either a TWG and/or NAC member. The purpose of this survey is to assess the process for HBP design, so this can be improved in the future.

The HBP process has largely been taking place through physical meetings but, because of Covid-19, the final NAC meeting was held online. In your responses in the survey, we like you to refer to the whole process, including physical and online meetings. At the end of the survey, you find a specific question on the online NAC meeting (June 2020).

Completing this survey will take approximately 10 - 15 minutes. All your answers will be treated confidentially: no attribution will be made to specific persons.

1. Please select your choice below. Clicking on the "agree" button below indicates that you have read the above information and you voluntarily agree to participate. If you do not wish to participate in the research study, please decline participation by clicking on the "disagree" button.

□ I agree

□ I disagree

Stakeholder involvement

2. Please indicate to what extent you agree with the following statements on stakeholder involvement on a scale of 1 (Strongly disagree) – 5 (Strongly agree).

	**Strongly disagree**	**Disagree**	**Neutral**	**Agree**	**Strongly agree**
It is clear to me how the stakeholders were selected to participate in the HBP design					
All important stakeholders were involved in the HBP design					
My involvement in the HBP design was valuable					
Involved stakeholders had equal opportunities to contribute during meetings					
Deliberation amongst stakeholders contributed to the development of my own opinions					
Views of involved stakeholders have been adequately taken into account in the HBP design					

3. How could involvement of stakeholders in HBP design be improved?

Decision criteria

4. Please indicate to what extent you agree with the following statements on decision criteria on a scale of 1 (Strongly disagree) – 5 (Strongly agree).

	**Strongly disagree**	**Disagree**	**Neutral**	**Agree**	**Strongly agree**
The criterion of “health gain for money spent” was clear to me					
The criterion of “avoidable burden of disease by the intervention” was clear to me					
The criterion of “budget impact” was clear to me					
The criterion of “feasibility” was clear to me					
The criterion of “equity” was clear to me					
The criterion of “social and economic impact” was clear to me					
The criterion of “financial risk protection” was clear to me					
The decision criteria are an adequate reflection of the most important values for HBP design					
The trade-offs between different criteria were clear to me					
Each criterion was adequately taken into account in the HBP design					

5. Are any decision criteria for HBP design missing?

6. How could the (use of) decision criteria for HBP design be improved?

Evidence

7. Please indicate to what extent you agree with the following statements on the use of evidence on a scale of 1 (Strongly disagree) – 5 (Strongly agree).

	**Strongly disagree**	**Disagree**	**Neutral**	**Agree**	**Strongly agree**
The evidence presented was clear to me					
There was sufficient time to understand the evidence on each intervention					
The evidence presented was relevant to design the HBP					
It is clear to me how the evidence was developed					
I am generally satisfied with the methods used to assess the evidence					
The evidence presented was sufficiently sensitive to the context of Pakistan					

8. How could the (use of) evidence for the development of the HBP be improved?

Appraisal process

9. Please indicate to what extent you agree with the following statements on the decision-process on a scale of 1 (Strongly disagree) – 5 (Strongly agree).

	**Strongly disagree**	**Disagree**	**Neutral**	**Agree**	**Strongly agree**
There was sufficient time to deliberate on each intervention					
Each intervention was evaluated according to the same standards					
The process for taking decisions about the inclusion of interventions into the HBP was clear to me					
I am satisfied with how decisions were taken about the inclusion of interventions in the HBP					
The interventions under discussion were relevant to the context of Pakistan					

10. How could the decision-process in the development of the HBP be improved?

General questions

11. Please indicate to what extent you agree with the following statements on a scale of 1 (Strongly disagree) – 5 (Strongly agree).
12.

	**Strongly disagree**	**Disagree**	**Neutral**	**Agree**	**Strongly agree**
The NAC meeting in June 2020 was organized online and this limited my understanding of the process					
The NAC meeting in June 2020 was organized online and this limited my involvement in the process					
The process and methods used have improved compared to previous approaches for HBP design in Pakistan					
The final content of the HBP is acceptable for the context of Pakistan					
I am satisfied with the outcomes of the HBP process					
The outcomes of the HBP process are relevant to my setting/area					
It is clear to me how the outcomes of the HBP process will be used moving forward					

13. If you wish to make any further comments about your experiences with the benefit package design in Pakistan and/or this survey, please use the space provided below

Personal information

14. Please indicate whether you participated in the TWG and/or the NAC
- □ TWG only
- □ NAC only
- □ TWG and NAC

15. What is your designation (position)?

16. What is the name of your organization/institution/department?

17. What province/federal area are you representing?
