# Supplementary material for: The Use of Evidence-Informed Deliberative Processes for Designing the Essential Package of Health Services in Pakistan
Source: Int J Health Policy Manag. 2023 Oct 24;12:8004. doi: 10.34172/ijhpm.2023.8004 (PMC10699818; doi:10.34172/ijhpm.2023.8004)
Supplement: Supplementary file 5 — Full Results of Priority Setting Process Evaluation Survey. [file ijhpm-12-8004-s005.pdf]

Article title: The Use of Evidence-Informed Deliberative Processes for Designing the Essential Package of Health Services in Pakistan

Journal name: International Journal of Health Policy and Management (IJHPM)

Authors' information: Rob Baltussen[1]*¶, Maarten Jansen[1]¶, Syeda Shehirbano Akhtar[2], Leon Bijlmakers[1], Sergio Torres-Rueda[3], Muhammad Khalid[4], Wajeeha Raza[5], Maryam Huda[6], Gavin Surgey[1], Wahaj Zulfiqar[4], Anna Vassall[3], Raza Zaidi[4], Sameen Siddiqi[6], Ala Alwan[7]

[1]Department of Health Evidence, Radboud University Medical Center, Nijmegen, The Netherlands.

[2]Department of Health Services Policy and Management, Arnold School of Public Health, University of South Carolina, Columbia, SC, USA.

[3]Department of Global Health & Development, London School of Hygiene and Tropical Medicine, London, UK.

[4]Ministry of National Health Services, Regulations and Coordination, Islamabad, Pakistan.

5Centre for Health Economics, University of York, York, UK.

[6]Department of Community Health Sciences, Aga Khan University, Karachi, Pakistan.

[7]DCP3 Country Translation Project, London School of Hygiene and Tropical Medicine, London, UK.

***Correspondence to:** Rob Baltussen; Email: Rob.Baltussen@Radboudumc.nl

Citation: Baltussen R, Jansen M, Akhtar SS, et al. The use of evidence-informed deliberative processes for designing the essential package of health services in Pakistan. Int J Health Policy Manag. 2023;12:8004. doi:10.34172/ijhpm.2023.8004

Supplementary file 5. Full Results of Priority Setting Process Evaluation Survey

The main text summarises the answers of respondents to the open-ended questions. Here we report more details.

Stakeholder involvement

Open Question: How could involvement of stakeholders in HBP design be improved?

Respondents mentioned stakeholder involvement in general could be improved by ensuring better inclusive recruitment of relevant stakeholders; involvement by the provinces; the private sector. More time for interaction between public health professionals and clinicians would also have been useful. In addition, stakeholder involvement could be improved by more timely sharing of documents for reviewing, providing more space and anonymity to stakeholders during meetings, stronger capacity building and improving continuing communication with stakeholders on steps

taken in-between meetings. With regard to the final meeting hosted online due to Covid-19, it was suggested smaller groups working to prepare the meeting might have been useful.

The (use of) decision criteria

Open question: Are any decision criteria for HBP design missing?

Respondents mention the decision criteria could be improved by additionally reflecting the health system costs, to what extent the private sector caters for an intervention, to what extent an intervention is currently already being provided, how it relates to the social health insurance program currently being implemented in the country and the complementarity of interventions.

Open question: How could the (use of) decision criteria for HBP design be improved?

Furthermore, participants mention more clear definitions of criteria could be provided and possibly illustrated with examples, e.g. with regard to the 'burden of disease avoided by the intervention' and 'budget impact'. The calculations used to score the performance of interventions on criteria could be explained more clearly. Ideally, criteria that lack evidence should be provided with some value(s) too. The use of evidence could also benefit from more timely sharing of background documents to allow familiarization with the decision criteria, better involvement of relevant departments and providing more information on the overall burden of disease and prevalence of conditions in Pakistan. Another participant mentioned 'feasibility' and 'utilization of shared resources' was not given due emphasis.

The (use of) evidence

Open question: How could the (use of) evidence for the development of the HBP be improved?

Respondents mention the (use of) evidence could be improved by the timely sharing of evidence sheets prior to meetings, the collection of local evidence, contacting of local public and private sector organizations to inquire about any ongoing work that they may be able to share, and updating some of the used definitions to better reflect clinical perspectives. Respondents also mention the use of evidence could be improved by providing more transparency regarding data sources and data collection methods.

The appraisal process

Open question: How could the appraisal process in the development of the HBP be improved?

Respondents mention that the appraisal process could be improved by increasing contributions from the provincial level (or even starting the process at the provincial level rather than national level) and those working on the ground; involving the relevant departments; and more thoughtful selection of relevant stakeholders to represent each cluster. Others mention they would like to see stronger engagement of health care providers and clinicians during the assessment of interventions.
